# Clinical Applications and Emerging Roles of Bone Wax in Orthopaedic Surgery: A Scoping Review

**DOI:** 10.3390/jcm15135226

**Published:** 2026-07-03

**Authors:** Ruijiang Li, Yimin Chen, Feng Gao, Chao Tu, Gang Liu, Jing Zhang, Minghui Yang

**Affiliations:** 1 Department of Orthopedics and Traumatology, Beijing Jishuitan Hospital, Beijing 100035, China; liruijiang017@163.com (R.L.); ymchen@pku.edu.cn (Y.C.); gaofeng@bjmu.edu.cn (F.G.); 13716013700@163.com (C.T.); liugangjst@163.com (G.L.); 2 Seventh Clinical Medical College, Capital Medical University, Beijing 100035, China; 3 National Center for Orthopaedics, Beijing 100035, China; 4 Fourth School of Clinical Medicine, Peking University, Beijing 100035, China; 5 School of Public Health, Harbin Medical University, Harbin 150081, China; jing.zhang@hrbmu.edu.cn

**Keywords:** bone wax, hemostasis, orthopaedic surgery, clinical applications, scoping review

## Abstract

**Background:** Perioperative bleeding from cancellous bone remains a clinically relevant challenge in orthopaedic surgery. Bone wax has long been used as a simple and inexpensive mechanical hemostatic agent, but previous reviews have focused mainly on total joint arthroplasty or on the material history of bone wax and its substitutes. The broader clinical evidence across orthopaedic subspecialties has not been comprehensively mapped. **Methods:** This scoping review followed the Arksey and O’Malley framework, with methodological refinements proposed by Levac et al., and was reported in accordance with PRISMA-ScR. Five electronic sources and grey literature were searched through May 2025. Clinical studies evaluating bone wax in orthopaedic surgery were eligible regardless of study design; a relevant clinical trial registry record with sufficient methodological detail was also retained to map ongoing evidence. Preclinical and purely material-based studies were excluded. The protocol was registered in the Open Science Framework (DOI: 10.17605/OSF.IO/K3ZAV). **Results:** Of 486 identified records, 15 met the inclusion criteria. Eight records concerned joint replacement, two spinal surgery, three excision of abnormal bony structures, and two sealing applications. The strongest comparative evidence came from arthroplasty, where bone wax was generally associated with reduced intraoperative or perioperative blood loss without clear evidence of adverse effects on early postoperative recovery outcomes reported in the included studies. Evidence for spinal and nontraditional applications was more heterogeneous and was derived mainly from small observational studies, case series, technical reports, or a trial registry record. Complications such as foreign-body reaction and chronic inflammation were described primarily in spine-related reports of retained nonabsorbable wax. **Conclusions:** Bone wax remains a useful and inexpensive adjunct for hemostasis in orthopaedic surgery. The strongest clinical evidence is concentrated in arthroplasty, whereas spinal and nontraditional applications remain supported by limited and heterogeneous data. Future research should clarify indication-specific benefits and risks and evaluate the safety and effectiveness of bioresorbable alternatives in well-designed clinical studies.

## 1. Introduction

Bleeding from cancellous bone after osteotomy or fracture-related bone preparation remains a common technical problem in orthopaedic surgery [1]. Owing to the porous architecture of cancellous bone and its limited capacity for intrinsic compression, substantial intraoperative blood loss may occur during major procedures, particularly reconstructive spine operations and arthroplasty [2,3,4,5,6,7]. Excessive surgical bleeding and transfusion can adversely affect recovery by increasing the risk of wound complications, infection, prolonged hospitalization, and higher costs [3,4,5,6,7]. Effective local hemostasis is therefore important in many orthopaedic settings.

Bone wax, composed primarily of beeswax and softening agents, acts as a mechanical barrier that tamponades bleeding from exposed cancellous bone surfaces [8]. Since its introduction in the late nineteenth century, it has been widely used because it is inexpensive, easy to apply, and capable of producing immediate local hemostasis. In orthopaedic practice, its use has been described most commonly in total knee and hip arthroplasty [9], but it has also been reported in spinal surgery [10,11,12]. At the same time, traditional nonabsorbable bone wax has raised concerns regarding foreign-body granuloma [13,14], chronic inflammation [15], infection [16], and interference with bone healing [17]. These limitations have stimulated interest in alternative absorbable formulations.

A scoping approach provides an appropriate framework for identifying, categorizing, and summarizing the available evidence, and is particularly suitable for mapping the breadth and diversity of evidence in a complex and heterogeneous field [18,19,20]. A conventional systematic review or meta-analysis was not considered appropriate at this stage because the clinical use of bone wax spans multiple orthopaedic subspecialties and does not rely on uniform surgical indications, comparator strategies, application techniques, outcome definitions, or follow-up durations. Moreover, the clinical role of bone wax extends beyond conventional cancellous-bone hemostasis to include barrier and sealing applications, which cannot be adequately addressed by a single effect-size question. Therefore, the present review was not designed to generate a pooled estimate of treatment efficacy, but to synthesize clinical evidence on the use of bone wax in orthopaedic surgery, highlight evolving applications, and identify priorities for future clinical research, particularly in relation to absorbable alternatives.

## 2. Methods

### 2.1. Study Design

This scoping review was conducted according to the methodological framework proposed by Arksey and O’Malley [19] and refined by Levac et al. [18]. Reporting followed the PRISMA extension for Scoping Reviews (PRISMA-ScR), and the completed PRISMA-ScR checklist is provided in Supplementary File S1. The protocol was registered in OSF Registries (DOI: 10.17605/OSF.IO/K3ZAV).

### 2.2. Research Question

The primary research question was: what clinical evidence is available regarding the use of bone wax in orthopaedic surgery, and how has its role expanded beyond conventional hemostasis?

### 2.3. Data Sources and Search Strategy

To maximize coverage of the literature, searches were conducted in five electronic databases: PubMed, Web of Science Core Collection, Scopus, Embase, and Ovid MEDLINE. All searches covered the period from database inception to 31 May 2025 and were limited to English-language human studies. Search terms combined controlled vocabulary and free-text words related to bone wax and orthopaedic procedures, including “bone wax”, “absorbable bone wax”, “resorbable bone wax”, “biodegradable bone wax”, “novel bone wax”, “orthopaedic surgery”, “orthopedic procedures”, “arthroplasty”, “joint replacement”, “arthroscopy”, “spine surgery”, and “fracture fixation”.

The database searches retrieved 486 records in total, including 49 records from PubMed, 233 from Embase, 50 from Ovid MEDLINE, 112 from Scopus, and 42 from Web of Science Core Collection. Grey literature was additionally searched through Google Scholar to capture relevant non-indexed clinical material. The first 100 Google Scholar records sorted by relevance were screened. No additional eligible studies were identified beyond the records already captured in the included evidence map. Manual reference checking of included studies and relevant reviews did not identify additional eligible studies. The complete search strategies are presented in Supplementary File S2.

### 2.4. Eligibility Criteria and Study Selection

Eligibility criteria were defined prospectively (Supplementary File S3). Clinical studies evaluating the use of bone wax in orthopaedic surgery were eligible regardless of study design. The registry record was not considered equivalent to a peer-reviewed clinical study. It was retained only when it described an orthopaedic bone-wax intervention with sufficient methodological detail to map ongoing clinical research. Registry information was used descriptively and was not used to infer effectiveness or safety. Randomized controlled trials, prospective cohort studies, case–control studies, retrospective studies, case series, technical notes, and the eligible registry record were considered. Conference abstracts, letters, annual reports, and book chapters were excluded because they did not provide sufficient detail for charting. Preclinical, animal, and purely material-development studies were also excluded.

Two reviewers independently screened titles and abstracts, followed by full-text assessment of potentially eligible records. Disagreements were resolved by discussion and, when necessary, consultation with a third reviewer. All identified records were managed in EndNote 21.

### 2.5. Data Charting

Data extraction was performed independently by the same two reviewers using a standardized charting form. Extracted items included author, year, country, journal/source, study design, sample size, orthopaedic indication, bone wax application details, comparator, reported outcomes, key findings, and study limitations. Data were charted in Microsoft Excel.

### 2.6. Synthesis of Results

As the purpose of a scoping review is to map evidence rather than estimate pooled effects, no formal meta-analysis was performed. Likewise, no formal risk-of-bias tool was applied. Instead, the included evidence was summarized descriptively according to orthopaedic subspecialty, type of application, study design, and reported outcome domains. To improve interpretability, we also summarized methodological and reporting characteristics of the included evidence.

## 3. Results

### 3.1. Literature Screening

**Figure 1:**
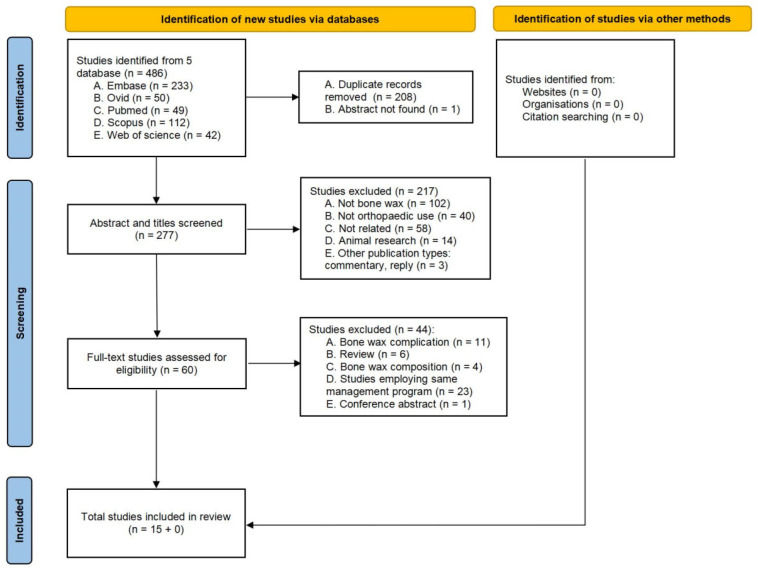
PRISMA-ScR flow diagram of the literature selection process.

A total of 486 records were identified across the database and grey-literature searches. After removal of duplicates and screening of titles and abstracts, potentially relevant full texts were assessed. Fifteen records met the inclusion criteria and were retained in the final evidence map. The study selection process is shown in Figure 1.

### 3.2. Methodological and Reporting Characteristics of the Included Evidence

Among the included records, six were published randomized controlled trials and one was a randomized trial registry record. In the published randomized trials, blood loss was consistently the principal endpoint, but outcome reporting was heterogeneous with respect to calculation methods and postoperative time points [21,22,23,24,25,26]. In one registry record, a randomized single-blinded design was described but peer-reviewed outcome data were not yet available [27]. This record was therefore interpreted only as evidence of ongoing clinical research activity and was not used to support any conclusion regarding the effectiveness or safety of bone wax.

Three studies used retrospective designs [28,29,30], one was a prospective case–control study [31], one was a prospective cohort study [32], two were case series [33,34], and one was a technical note [35]. Several non-arthroplasty reports were limited by small sample sizes, highly selected indications, or lack of quantitative comparative outcome data [29,30,32,33,34,35]. These features underscore that the evidence base is methodologically uneven across indications, with comparatively stronger comparative data in arthroplasty than in other settings.

### 3.3. Study Characteristics

There was a year-on-year increase in publications addressing the clinical use of bone wax. The included records were conducted across nine countries, with four from China [21,22,24,31], three from the United States [27,30,32], two from Japan [29,34], and one each from France [33], Belgium [35], Singapore [23], South Korea [28], Turkey [25], and Iran [26].

Among the 15 included records, eight concerned joint replacement procedures (five total knee arthroplasty [21,23,24,27,28], one unicompartmental knee arthroplasty [31], and two total hip arthroplasty [22,26]), two addressed spinal surgery [25,29], three involved the excision of abnormal bony structures [30,32,34], and two described sealing applications [33,35]. A detailed summary of the included evidence is presented in Table 1.

### 3.4. Joint Replacement

The application of bone wax in joint replacement has been reported predominantly during the past decade, and this subgroup contains the strongest comparative evidence. Across knee and hip arthroplasty, bone wax was generally associated with reduced intraoperative or perioperative blood loss, with no clear signal of impaired early postoperative recovery outcomes in the included reports [22,23,24,26,27,28,31]. These findings suggest that bone wax may be a useful adjunct for hemostasis during arthroplasty, although comparator strategies and blood-loss measurements varied across studies.

### 3.5. Knee Arthroplasty

A double-blind randomized controlled trial conducted in Singapore compared bone wax with electrocautery during total knee arthroplasty and found significantly lower perioperative blood loss in the bone wax group [23]. Similar findings were reported in subsequent studies from China and South Korea [24,28]. A recent prospective case–control study in unicompartmental knee arthroplasty also found lower total blood loss with bone wax than without bone wax [31]. In addition, a randomized trial registry record suggested continued interest in this question for primary unilateral total knee arthroplasty, although peer-reviewed results were not yet available [27]. By contrast, an older randomized study reported less blood loss with a tourniquet-based strategy than with bone wax plus electrocautery, indicating that the effect of bone wax depends on the broader blood-management protocol [21].

### 3.6. Hip Arthroplasty

Clinical evidence in hip arthroplasty remains limited to two randomized controlled trials, one from China and one from Iran [22,26]. Both studies reported lower perioperative or early postoperative blood loss in the bone wax groups than in the control groups. Taken together, the available evidence suggests that bone wax may contribute meaningfully to local hemostasis during total hip arthroplasty, but the evidence base is still modest in size.

### 3.7. Spinal Surgery

Published evidence for spinal applications is less extensive than for arthroplasty. In a randomized trial from Turkey, bone wax reduced postoperative drainage compared with other hemostatic approaches when used at the iliac crest donor site during spinal surgery [25]. More recently, Inoue et al. described a nozzle-based technique for endoscopic bone wax delivery, emphasizing precise local hemostasis and improved visualization during full-endoscopic lumbar laminotomy [29]. These reports suggest potential utility in spine surgery, but the current evidence remains indication-specific and methodologically limited.

### 3.8. Excision of Abnormal Bony Structures

Bone wax has also been used deliberately as a local barrier in procedures involving the excision of abnormal bony structures. A retrospective comparative study reported lower re-ossification rates after calcaneonavicular coalition resection when bone wax or autologous fat was used as an interposition material, compared with extensor digitorum brevis interposition [30]. Case-based reports further described its use after excision of distal tibial bony bridges [34] and in post-traumatic radioulnar synostosis or heterotopic ossification [32]. In these settings, the nonabsorbable nature of traditional bone wax, which is often viewed as a limitation, was intentionally exploited as a barrier to recurrent bone formation.

### 3.9. Other Innovative Applications

Two small reports described sealing-related applications beyond conventional hemostasis. Bone wax was used to reduce blood leakage through cannulated screws during arthroscopic anterior cruciate ligament reconstruction [35], and it was also reported to improve the seal and vacuum efficiency of negative-pressure wound therapy dressings [33]. These applications are innovative, but the supporting evidence is limited to small descriptive reports.

## 4. Discussion

This scoping review mapped 15 clinical records on the use of bone wax in orthopaedic surgery and showed that the evidentiary strength is highly indication-specific. Arthroplasty accounts for the largest and methodologically strongest subgroup, whereas evidence for spinal and nontraditional applications remains sparse, heterogeneous, and often based on small observational reports, case series, technical notes, or a registry record. The main contribution of this review is therefore not simply to reaffirm that bone wax can assist hemostasis, but to show that its clinical support is unevenly distributed across orthopaedic subspecialties. This distinction is clinically important because bone wax is often regarded as a familiar and low-cost material, yet its risk–benefit profile may differ substantially depending on the anatomical site, amount applied, and whether the intended role is hemostasis, barrier formation, or sealing.

### 4.1. Bone Wax in Arthroplasty

Perioperative blood loss has important consequences in joint replacement, including postoperative anaemia, transfusion, delayed recovery, and increased healthcare utilization [3,4,5,6,7,36,37,38,39]. Bone wax is particularly suited to controlling bleeding from exposed cancellous bone and osteotomy surfaces [39]. A recent meta-analysis focused on total joint arthroplasty found that bone wax reduced transfusion requirements, blood loss, and haemoglobin decline [40]. However, that meta-analysis included only three studies. The present scoping review extends that observation by mapping eight records from knee and hip arthroplasty, including six published randomized trials, one prospective case–control study, and one registry record. Overall, the arthroplasty literature provides the most consistent comparative support for the hemostatic effect of bone wax. Studies included in this scoping review varied in blood-loss definitions, comparator strategies, perioperative protocols, and follow-up duration. Therefore, the most defensible interpretation is that bone wax appears to be a useful local adjunct in selected arthroplasty procedures, but its incremental value should be evaluated within multimodal blood management.

Tranexamic acid (TXA) is now widely used as a pharmacologic blood-conservation strategy in arthroplasty [41,42]. Although TXA acts as an antifibrinolytic agent, available clinical experience in arthroplasty has not demonstrated a significant increase in venous thromboembolism when TXA is used according to standard perioperative protocols. This interpretation is also supported by recent hematologic evidence showing that plasminogen activation and plasmin activity are not necessary to prevent venous thrombosis or thromboembolism [43], as well as by the accompanying commentary by Yee emphasizing that antifibrinolysis should not be equated with thrombus initiation [44]. Therefore, future studies of bone wax in arthroplasty should clarify whether topical mechanical hemostasis provides incremental benefit when used alongside contemporary TXA-based multimodal blood-management pathways.

### 4.2. Bone Wax in Spinal Surgery

Bone bleeding may be technically demanding during spinal surgery, particularly in reconstructive procedures and operations performed through narrow working corridors [10]. Existing literature has long recognized bone wax as a practical aid for osseous bleeding control in these settings. Evans et al. proposed a novel “sausage-shaped” technique in which bone wax was molded and inserted into a 1 mL syringe. By gently pressing the syringe plunger, bone wax could be delivered precisely into bleeding cancellous bone, effectively sealing the site and achieving hemostasis during the anterior cervical discectomy and fusion (ACDF) operation [45]. In the studies included here, bone wax was associated with reduced postoperative drainage in a randomized spinal-surgery trial [25], and newer endoscopic delivery methods appeared to facilitate precise local hemostasis in minimally invasive procedures [29].

At the same time, spinal surgery is the setting in which complications related to retained nonabsorbable bone wax have been most clearly described. The effective mechanical barrier of bone wax may become problematic when excessive material is retained in confined anatomical spaces, especially in spinal surgery. Case reports have linked retained bone wax to foreign-body reaction, epidural mass formation, and neural compression [13,46]. These reports do not negate its hemostatic value, but they do emphasize the need for restrained use, precise placement, and avoidance of unnecessary material in confined spaces. The balance between efficacy and safety in spinal procedures remains insufficiently defined.

### 4.3. Bone Wax in Other Clinical Uses

Complications associated with bone wax application have been reported, largely due to its composition and physicochemical characteristics. Because bone wax acts as a nonabsorbable foreign material, it may trigger chronic inflammatory responses and interfere with bone healing. One of the more interesting findings of this review is that a property often regarded as a disadvantage—the persistence of traditional nonabsorbable bone wax—has been deliberately exploited in selected procedures. In resection of coalition, bony bridge, or heterotopic bone, bone wax has been used as an interposition barrier to limit recurrent ossification [30,32,34,47]. In these indications, delayed incorporation is not merely tolerated but may be therapeutically useful. This suggests that the clinical value of bone wax is context-dependent: in procedures where bone healing is desired, persistence may be disadvantageous, whereas in procedures where re-ossification is the problem, persistence may be beneficial. Similarly, the sealing characteristics of bone wax have enabled procedural innovations beyond classical bone-surface hemostasis. The included reports suggest potential value in sealing cannulated screw channels and improving the seal of negative-pressure systems [33,35]. However, these applications remain supported by very limited clinical evidence and should be interpreted cautiously. Future studies should determine whether these techniques could reduce clinically meaningful outcomes such as postoperative hematoma, dressing failure, air leakage, and wound complications.

### 4.4. Toward Absorbable Bone Wax

The formal evidence mapped in this review was restricted to clinical studies and one relevant registry record; preclinical and purely material-development investigations were intentionally excluded. Nevertheless, recent biomaterials research has produced several candidate substitutes that aim to preserve hemostatic performance while mitigating the limitations of nonabsorbable wax. Experimental formulations containing copper, calcium-based components, modified starch, or β-tricalcium phosphate have shown promising hemostatic, antibacterial, angiogenic, or osteogenic properties in preclinical studies [48,49,50]. These developments reflect a shift in the design goal of bone wax substitutes: the ideal material should not only stop bleeding but also degrade predictably, avoid persistent foreign-body reaction, reduce infection risk, and allow or even promote bone regeneration.

Feng et al. proposed a novel bioresorbable bone wax based on a polymer dispersion matrix incorporating quaternized cationic starch and β-tricalcium phosphate, aiming to achieve immediate control of cancellous bone bleeding while supporting subsequent material resorption and bone regeneration [51]. Early clinical evidence is also beginning to emerge. A pilot clinical trial of water-soluble Tableau wax in lumbar fusion suggested lower perioperative blood loss than conventional management [52]. More recently, Wu et al. reported a prospective, randomized, single-blinded, positive-controlled trial comparing a novel absorbable bone wax with conventional bone wax in patients undergoing periacetabular osteotomy [53]. The study reported comparable hemostasis time between groups, no apparent short-term wound-healing concerns, and postoperative imaging findings, suggesting absorbability of the novel bone wax without impairment of osteotomy healing. Because this study was published after the search window of the present scoping review, it was discussed as recent post-search clinical evidence rather than incorporated into the formal evidence map.

These developments are encouraging, but broader clinical adoption will require rigorous comparative trials and longer-term safety evaluation. Future studies should evaluate absorbable bone wax across arthroplasty, spinal surgery, fracture surgery, and osteotomy procedures, with standardized outcomes including hemostasis time, perioperative blood loss, transfusion, local inflammatory reaction, infection, radiographic bone healing, and material-related adverse events.

### 4.5. Evidence Quality and Interpretability

The apparent hemostatic benefit of bone wax should be interpreted in light of several methodological limitations. Even within arthroplasty, where comparative evidence is most concentrated, several studies had relatively small sample sizes, and blood loss was reported using different definitions, including intraoperative blood loss, drainage volume, calculated blood loss, total blood loss, and hemoglobin decline. Follow-up was generally short, limiting the ability to evaluate delayed complications such as foreign-body reaction, infection, impaired bone healing, or granuloma formation. Adverse-event reporting was also inconsistent across studies, and most reports were not designed or powered to detect uncommon material-related complications.

Outside arthroplasty, this scoping review identified several additional orthopaedic applications of bone wax and summarized their main clinical characteristics. However, the quality of evidence supporting these applications remains relatively low. Most non-arthroplasty reports were retrospective studies, case series, or technical notes, and high-quality comparative clinical studies are still lacking. As a result, current evidence is insufficient to robustly confirm the hemostatic effectiveness or other proposed procedural benefits of bone wax in these orthopaedic settings. These limitations do not negate the potential clinical usefulness of bone wax, but reduce confidence in broad claims of effectiveness and safety across all orthopaedic settings.

This review also identified emerging evidence on absorbable bone wax. As a new generation of local hemostatic material, absorbable bone wax is expected not only to provide reliable hemostatic performance but also to degrade predictably and support bone regeneration. However, most available studies on absorbable formulations remain at the preclinical or early clinical stage, and their findings have not yet been supported by sufficient clinical evidence. As an important future direction for the development of bone wax substitutes, clinical validation of the effectiveness, safety, degradation behavior, and bone-healing compatibility of absorbable bone wax is likely to become a key focus in this field.

### 4.6. Strengths and Limitations

This review has several strengths. It comprehensively mapped the clinical use of bone wax in orthopaedic surgery and showed that its applications extend across multiple orthopaedic subspecialties. The available evidence was not limited to intraoperative hemostasis in arthroplasty, but also included endoscopic hemostatic techniques in spinal surgery and nontraditional uses that take advantage of the stable physical properties of conventional bone wax. These included sealing applications and interposition or packing strategies after procedures such as bony bridge resection. By summarizing these diverse clinical scenarios, this review highlights that the value of bone wax is context-dependent and may differ according to whether the intended role is hemostasis, sealing, or prevention of recurrent ossification. In addition, this review discussed recent developments in absorbable bone wax, providing a clinical and conceptual basis for future studies evaluating the effectiveness, safety, degradation behavior, and bone-healing compatibility of next-generation absorbable formulations.

Several limitations should also be acknowledged. First, the review was restricted to English-language records, and relevant studies in other languages may therefore have been missed. Second, because this was a scoping review, no formal risk-of-bias tool was applied and no quantitative pooling was attempted. Third, the literature remains methodologically uneven: many non-arthroplasty applications were described only in small observational studies, case series, technical notes, or a registry record without peer-reviewed outcome publication. Finally, the clinical evidence on newer absorbable formulations remains limited, so conclusions regarding their comparative effectiveness should remain cautious.

## 5. Conclusions

Bone wax remains a widely used and effective adjunct for hemostasis in orthopaedic surgery. The currently available clinical evidence suggests that its hemostatic benefit is most consistently supported in arthroplasty, particularly in knee and hip replacement procedures. In contrast, evidence for spinal surgery, barrier applications, sealing techniques, and other nontraditional uses remains limited, heterogeneous, and generally lower in methodological quality. Future research should refine indication-specific use, optimize application techniques, and evaluate absorbable alternatives in well-designed clinical studies.

## Figures and Tables

**Table 1:** Clinical evidence on the application of bone wax in orthopaedic surgery.

Study	Design and Sample	Surgery	Bone-Wax and Comparator	Outcome	Main Finding
Ye 2024 [24]	RCT; *n* = 150	TKA	Cancellous surfaces; timing strategies vs. no bone wax	Blood loss	Repeated application reduced perioperative blood loss.
Shi 2024 [31]	Prospective case–control *n* = 116	UKA	Exposed cancellous surfaces vs. no bone wax	Blood loss; complications	Lower total blood loss without apparent short-term complication increase.
Li 2023 [22]	RCT; *n* = 104	THA	Exposed cancellous bone vs. no bone wax	Blood loss	Reduced early postoperative blood loss on POD1 and POD3.
Mortazavi 2022 [26]	RCT; *n* = 152	THA	Exposed cancellous surfaces vs. no bone wax	Blood loss	Reduced apparent and calculated perioperative blood loss.
Segal 2021 [27]	Registry record target *n* = 100; enrolled *n* = 52	TKA	After cemented implants vs. no bone wax	Blood loss; safety; function	Ongoing randomized comparison described; no peer-reviewed results available.
Shin 2020 [28]	Retrospective comparative *n* = 62	TKA	Intraoperative hemostasis vs. no bone wax	Blood loss; transfusion	Associated with reduced blood loss and fewer transfusions.
Moo 2017 [23]	RCT; *n* = 100	TKA	Intraoperative hemostasis vs. electrocautery	Blood loss; complications	Reduced total perioperative blood loss; no wax-related complications reported during short follow-up.
Guo 2013 [21]	RCT; *n* = 60	TKA	Bone wax plus electrocautery vs. tourniquet strategy	Blood loss; drainage	Tourniquet strategy resulted in less blood loss than bone wax plus electrocautery.
Çopuroğlu 2011 [25]	RCT; *n* = 66	Spinal fusion / iliac crest donor site	Donor-site hemostasis vs. gelatin sponge or control	Drainage	Reduced postoperative drainage compared with comparator groups.
Inoue 2023 [29]	Retrospective technical report	Full-endoscopic lumbar laminotomy	Nozzle-based endoscopic application; no comparator	Hemostasis; visualization	Practical focal osseous bleeding control and visualization were described.
Masquijo 2017 [30]	Retrospective comparative 80 feet	Calcaneonavicular coalition resection	Interposition barrier vs. fat graft or extensor digitorum brevis	Re-ossification; function	Bone wax and fat grafting were associated with lower re-ossification.
Yoshida 2008 [34]	Case series; *n* = 4	Distal tibial partial growth arrest	Packing after bony bridge resection; no comparator	Recurrence	Reported to help prevent bony bridge re-formation.
Kamineni 2002 [32]	Prospective cohort; *n* = 7	Proximal radial resection	Interposition barrier after resection; no comparator	Function; recurrence	Technique appeared useful for improving rotation and limiting re-ossification.
Bulla 2014 [33]	Case series; *n* = 2	Negative-pressure wound therapy	Dressing seal enhancement; no comparator	Sealing efficiency	Improved dressing seal and vacuum efficiency in reported cases.
Bohy 2002 [35]	Technical note	Arthroscopic ACL reconstruction	Cannulated screw-channel sealing; no comparator	Hematoma prevention	Proposed to reduce blood leakage through cannulated screws.

Abbreviations: RCT: randomized controlled trial; POD: post operative day; THA, total hip arthroplasty; TKA, total knee arthroplasty; UKA, unicompartmental knee arthroplasty; ACL: Anterior Cruciate Ligament.

## Data Availability

All data generated or analyzed during this study are included in this published article and its Supplementary Information Files. Further information is available from the corresponding author on reasonable request.

## Supplementary Materials

The following supporting information can be downloaded at: https://www.mdpi.com/article/10.3390/jcm15135226/s1, Supplementary File S1. PRISMA-ScR-Fillable-Checklist; Supplementary File S2. Literature Searching Process; Supplementary File S3. Inclusion and Exclusion Criteria.

## Abbreviations

OSF	Open Science Framework
PRISMA-ScR	Preferred Reporting Items for Systematic Reviews and Meta-Analyses Extension for Scoping Reviews
RCT	randomized controlled trial
POD	post operative day
THA	total hip arthroplasty
TKA	total knee arthroplasty
TXA	tranexamic acid
UKA	unicompartmental knee arthroplasty
ACL	Anterior Cruciate Ligament
