# Supplementary material for: Clinical Applications and Emerging Roles of Bone Wax in Orthopaedic Surgery: A Scoping Review
Source: J Clin Med. 2026 Jul 3;15(13):5226. doi: 10.3390/jcm15135226 (PMC13363597; doi:10.3390/jcm15135226)
Supplement: Supplementary file 1 [file jcm-15-05226-s001.zip › jcm-4358662-supplementary/Supplementary File S1. PRISMA-ScR-Fillable-Checklist.pdf]

Preferred Reporting Items for Systematic reviews and Meta-Analyses extension for Scoping Reviews (PRISMA-ScR) Checklist

SECTION	ITEM	PRISMA-ScR CHECKLIST ITEM	REPORTED ON PAGE #
**TITLE**			
Title	1	Identify the report as a scoping review.	Application of Bone Wax in Orthopaedic Surgery: A Scoping Review
**ABSTRACT**			
Structured summary	2	Provide a structured summary that includes (as applicable): background, objectives, eligibility criteria, sources of evidence, charting methods, results, and conclusions that relate to the review questions and objectives.	The abstract includes a concise background outlining the clinical significance of bone wax in orthopaedic surgery and the rationale for conducting a scoping review. The objectives clearly state the intent to map and summarize current evidence regarding bone wax composition, clinical applications, and associated complications. Eligibility criteria specify inclusion of peer-reviewed clinical studies involving bone wax use in orthopaedic procedures, with exclusion of preclinical and purely material-based investigations. Sources of evidence include PubMed, Embase, Scopus, Ovid, and Web of Science, with supplementary searches in Google Scholar for grey literature. The charting methods describe data extraction and synthesis following PRISMA-ScR guidelines. Results summarize 16 included studies categorized by surgical type (arthroplasty, spine, bone resection) and evidence level. The conclusions address current clinical applications, existing limitations, and future directions for bioresorbable bone wax development, directly aligning with the review objectives.
**INTRODUCTION**			
Rationale	3	Describe the rationale for the review in the context of what is already known. Explain why the review questions/objectives lend themselves to a scoping review approach.	The introduction outlines the long-standing use of bone wax as a haemostatic material in orthopaedic surgery and highlights the increasing diversity of formulations and applications. Despite its widespread use, evidence regarding its clinical efficacy, complications, and evolving composition remains fragmented across various subspecialties. These gaps in knowledge justify a scoping review approach, as the topic involves heterogeneous study designs, diverse surgical contexts, and an emerging body of literature that cannot be synthesized quantitatively. The rationale explicitly explains that a

St. Michael's
Inspired Care.
Inspiring Science.

SECTION	ITEM	PRISMA-ScR CHECKLIST ITEM	REPORTED ON PAGE #
			scoping review allows for mapping the breadth of existing evidence, identifying key concepts, and highlighting areas requiring further research.
Objectives	4	Provide an explicit statement of the questions and objectives being addressed with reference to their key elements (e.g., population or participants, concepts, and context) or other relevant key elements used to conceptualize the review questions and/or objectives.	The objectives are clearly stated at the end of the Introduction section: to systematically map the evolution, composition, and clinical applications of bone wax in orthopaedic surgery; to summarize the existing evidence on its haemostatic effectiveness and safety; and to identify research gaps and future directions, particularly regarding the development and clinical translation of bioresorbable bone wax materials.
**METHODS**			
Protocol and registration	5	Indicate whether a review protocol exists; state if and where it can be accessed (e.g., a Web address); and if available, provide registration information, including the registration number.	A review protocol was developed in accordance with the PRISMA-ScR guidelines and prospectively registered in the **Open Science Framework (OSF)** under the registration number **10.17605/OSF.IO/K3ZAV**. The protocol details the objectives, eligibility criteria, search strategy, and data charting process for this scoping review. The full registration record is publicly accessible through the OSF platform.
Eligibility criteria	6	Specify characteristics of the sources of evidence used as eligibility criteria (e.g., years considered, language, and publication status), and provide a rationale.	Studies were eligible if they reported the clinical use of bone wax in orthopaedic surgery, regardless of study design. Only **peer-reviewed, English-language clinical studies** were included. The **search period extended from database inception to May 2025** to ensure comprehensive coverage of contemporary and historical literature. Exclusion criteria included **non-orthopaedic applications**, **duplicate reports**, **conference abstracts**, **letters**, **technical papers**, **book chapters**, and **preclinical or purely material-based investigations**. The inclusion and exclusion criteria were defined to ensure the review focused on clinically relevant evidence while maintaining methodological consistency for subsequent data charting.
Information sources*	7	Describe all information sources in the search (e.g., databases with dates of coverage and contact with authors to identify additional sources), as well as the date the most recent search was executed.	The literature search was conducted across five major electronic databases: **PubMed, Embase, Scopus, Ovid Journals, and Web of Science**, covering all records from **database inception to May 2025**. To enhance comprehensiveness, **grey literature** was

SECTION	ITEM	PRISMA-ScR CHECKLIST ITEM	REPORTED ON PAGE #
			additionally searched using **Google Scholar**. The search was last updated in **May 2025**. Reference lists of all included studies and relevant reviews were manually screened to identify any additional eligible publications.
Search	8	Present the full electronic search strategy for at least 1 database, including any limits used, such that it could be repeated.	A comprehensive search strategy was developed using a combination of controlled vocabulary (e.g., MeSH terms) and free-text keywords related to bone wax and orthopaedic surgery. The primary search terms included **"bone wax," "orthopaedic surgery,"** and their synonyms. The detailed search expressions for each database (PubMed, Embase, Ovid, Scopus, and Web of Science) are provided in **Appendix 1** to ensure reproducibility. The search strategy was peer reviewed within the author team and refined to optimize sensitivity and specificity. No restrictions were applied to study design, while the search was limited to **English-language publications** from **database inception to May 2025**.
Selection of sources of evidence†	9	State the process for selecting sources of evidence (i.e., screening and eligibility) included in the scoping review.	All records identified through database and grey literature searches were imported into EndNote for reference management, and duplicates were removed. Titles and abstracts were independently screened by two reviewers (**RL and YC**) according to predefined eligibility criteria. Full texts of potentially relevant articles were then retrieved and assessed for inclusion. Disagreements between reviewers were resolved through discussion and consultation with a third senior reviewer (**YM**). The selection process followed PRISMA-ScR guidelines, and a detailed flow diagram illustrating study identification, screening, eligibility, and inclusion is presented in **Figure 1.**
Data charting process‡	10	Describe the methods of charting data from the included sources of evidence (e.g., calibrated forms or forms that have been tested by the team before their use, and whether data charting was done independently or in duplicate) and any processes for obtaining and confirming data from investigators.	Data from the included studies were charted independently by two reviewers (**RL and YC**) using a standardized data extraction form developed by the research team. Extracted variables included study characteristics (authors, year, country, design, sample size), surgical type, main outcomes, and key findings. The charted data were cross-checked for accuracy, and any discrepancies were resolved through

SECTION	ITEM	PRISMA-ScR CHECKLIST ITEM	REPORTED ON PAGE #
			discussion or consultation with the senior investigator (**YM**). The final charted data were summarized in tabular format (**Table 1**) and used for narrative synthesis.
Data items	11	List and define all variables for which data were sought and any assumptions and simplifications made.	Data extracted from each included study comprised the following variables: **Bibliographic information:** author(s), year of publication, and country of origin; **Study characteristics:** study design, sample size, surgical type, and orthopaedic subspecialty; **Outcome measures:** perioperative blood loss, haemostatic effectiveness, complications, follow-up duration, and other reported outcomes related to bone wax use; **Key findings and conclusions.** Any unclear or missing data were noted and discussed among reviewers, with consensus reached through consultation with the senior investigator (**YM**). All data items were summarized in **Table 1**, which provides an overview of study characteristics and outcomes.
Critical appraisal of individual sources of evidence§	12	If done, provide a rationale for conducting a critical appraisal of included sources of evidence; describe the methods used and how this information was used in any data synthesis (if appropriate).	Although formal risk of bias assessment is not required for scoping reviews, a **descriptive quality appraisal** was conducted to evaluate the methodological robustness of the included studies, given the heterogeneity in study design. The appraisal focused on the clarity of study design, reporting of baseline comparability, specification of randomization methods, completeness of outcome reporting, and adequacy of follow-up. Among the seven randomized controlled trials, all declared no conflicts of interest and provided baseline data, though some lacked detailed descriptions of randomization or blood loss measurement methods. Similar observations were made for retrospective and prospective studies. The findings from this appraisal were summarized narratively within the *Results* section to contextualize evidence strength but were **not used for exclusion or weighting of studies**.
Synthesis of results	13	Describe the methods of handling and summarizing the data that were charted.	The charted data were synthesized descriptively and organized according to surgical category, study design, and reported outcomes. Results were summarized in tabular form (**Table 1**)

St. Michael's
Inspired Care.
Inspiring Science.

SECTION	ITEM	PRISMA-ScR CHECKLIST ITEM	REPORTED ON PAGE #
			and complemented by a narrative synthesis to highlight similarities, differences, and emerging patterns across studies. Evidence was grouped into four main application areas of bone wax: **joint arthroplasty**, **spinal surgery**, **abnormal bony structure resection**, and **other innovative uses**. Quantitative pooling was not conducted, as this review aimed to map rather than aggregate evidence. The synthesis emphasized the haemostatic effectiveness, complications, and clinical trends associated with bone wax use in orthopaedic practice.
**RESULTS**			
Selection of sources of evidence	14	Give numbers of sources of evidence screened, assessed for eligibility, and included in the review, with reasons for exclusions at each stage, ideally using a flow diagram.	A total of 486 **records** were identified through database and grey literature searches. After removal of duplicates, **277 titles and abstracts** were screened for relevance. **60 full-text articles** were assessed for eligibility, of which **16 studies** met the inclusion criteria and were included in the final synthesis. Reasons for exclusion at the full-text stage included: studies unrelated to orthopaedic applications, absence of clinical data, duplication, or being preclinical/material-based only. The full selection process, including numbers at each stage, is detailed in the **PRISMA flow diagram (Figure 1)**.
Characteristics of sources of evidence	15	For each source of evidence, present characteristics for which data were charted and provide the citations.	The characteristics of all included studies are summarized in **Table 1**. Key information extracted included authors, year of publication, country, study design, surgical type, sample size, main outcome measures, and key findings. The included studies covered a diverse range of orthopaedic subspecialties, including joint arthroplasty, spinal procedures, and resection of abnormal bony structures. Geographically, studies originated from **China (5), the United States (3), Japan (2), and one each from France, Belgium, Singapore, South Korea, and Iran**, reflecting the global distribution of research interest in bone wax applications. The included studies comprised **7 randomized controlled trials, 1 prospective cohort study, 1 case–control study, 4 retrospective studies, 2 case series, and 1 technical report**.

SECTION	ITEM	PRISMA-ScR CHECKLIST ITEM	REPORTED ON PAGE #
Critical appraisal within sources of evidence	16	If done, present data on critical appraisal of included sources of evidence (see item 12).	
Results of individual sources of evidence	17	For each included source of evidence, present the relevant data that were charted that relate to the review questions and objectives.	Findings from the 16 included studies are presented in Table 1 and detailed narratively in the Results section. Evidence was organized by orthopaedic subspecialty to align with the review objectives: **Joint arthroplasty:** Bone wax demonstrated consistent haemostatic efficacy in total knee and hip replacement, significantly reducing perioperative blood loss without adversely affecting wound healing or functional recovery. **Spinal surgery:** Studies reported bone wax as a safe and effective agent for controlling osseous bleeding during spinal fusion and endoscopic procedures, though isolated complications related to local compression or foreign body reaction were noted. **Abnormal bone structure resection:** Case reports and small series indicated bone wax could prevent reformation of bony bridges or heterotopic ossification by acting as a physical barrier. **Other clinical uses:** Applications included preventing bone cement leakage and improving vacuum sealing efficiency in negative pressure systems. These findings directly address the review questions by mapping the clinical roles, benefits, and limitations of bone wax in orthopaedic surgery.
Synthesis of results	18	Summarize and/or present the charting results as they relate to the review questions and objectives.	The synthesis revealed that bone wax remains a widely used and effective haemostatic material in orthopaedic surgery, with diverse clinical applications and evolving formulations. Across studies, bone wax consistently reduced perioperative blood loss in **joint arthroplasty**, particularly in total knee and hip replacement, without increasing postoperative complications or impairing wound or bone healing. In **spinal surgery**, bone wax effectively controlled osseous bleeding during fusion and endoscopic procedures, although a few reports noted rare complications such as local compression and foreign body

SECTION	ITEM	PRISMA-ScR CHECKLIST ITEM	REPORTED ON PAGE #
			reactions. In **abnormal bone resection**, its barrier function was successfully applied to prevent reformation of bony bridges and heterotopic ossification. Furthermore, several **innovative uses**—including prevention of bone cement leakage and enhancement of vacuum sealing—highlight the material's versatility in modern orthopaedics.<br>The results collectively address the review objectives by mapping the current clinical landscape of bone wax use, identifying common advantages and limitations, and emphasizing its evolving role in both established and emerging surgical contexts.
**DISCUSSION**			
Summary of evidence	19	Summarize the main results (including an overview of concepts, themes, and types of evidence available), link to the review questions and objectives, and consider the relevance to key groups.	The discussion integrates the evidence to provide a comprehensive overview of bone wax's clinical utility, safety, and evolving development. The synthesis highlights three key themes:<br>**Haemostatic efficacy and safety:** Evidence from randomized controlled trials confirms that bone wax effectively reduces perioperative bleeding in arthroplasty and spinal surgery, with few adverse effects on bone healing or function.<br>**Clinical heterogeneity and usage patterns:** Applications vary among orthopaedic subspecialties, reflecting differences in surgical exposure, bleeding control needs, and material handling. Joint arthroplasty appears to be the most extensively studied and reliable setting.<br>**Innovation and translational potential:** Recent developments in **bioresorbable bone wax** formulations demonstrate comparable haemostatic performance with improved biocompatibility, offering promising directions for clinical translation and future biomaterial innovation.<br>These findings collectively fulfill the scoping review objectives by mapping existing evidence, clarifying the strengths and limitations of current clinical practices, and identifying research gaps that warrant further investigation—particularly regarding long-term safety, optimized formulation, and comparative efficacy with pharmacologic haemostatic

SECTION	ITEM	PRISMA-ScR CHECKLIST ITEM	REPORTED ON PAGE #
			agents such as tranexamic acid.
Limitations	20	Discuss the limitations of the scoping review process.	This scoping review has several limitations inherent to its design. First, the review included only **English-language, peer-reviewed clinical studies**, which may have introduced language and publication bias. Second, as **preclinical and purely material-based investigations** were excluded, emerging experimental data on novel bioresorbable bone waxes were not captured, potentially limiting the comprehensiveness of material innovation analysis. Third, due to the heterogeneity of study designs, surgical contexts, and outcome measures, **quantitative synthesis was not performed**, and the conclusions are based on descriptive interpretation. Finally, the small sample sizes and limited number of high-quality trials restrict the strength of clinical inference. Nevertheless, the study provides a broad and structured overview of current clinical applications and research trends, which can inform future targeted investigations.
Conclusions	21	Provide a general interpretation of the results with respect to the review questions and objectives, as well as potential implications and/or next steps.	This scoping review comprehensively maps the current evidence on the clinical use of bone wax in orthopaedic surgery. Bone wax remains an effective and practical haemostatic agent, especially in **joint arthroplasty** and **spinal surgery**, demonstrating consistent blood loss reduction without increasing postoperative complications. Its physicochemical properties also enable innovative applications, such as preventing bone cement leakage and serving as a barrier to aberrant bone formation. However, limitations related to biocompatibility and delayed bone healing highlight the need for continued research. Emerging **bioresorbable bone wax formulations** show promising potential to combine effective haemostasis with improved biological integration. Future studies should focus on high-quality clinical trials and translational work to validate these materials and optimize their use across orthopaedic subspecialties.
**FUNDING**			
Funding	22	Describe sources of funding for	This work was supported by **Capital's**

SECTION	ITEM	PRISMA-ScR CHECKLIST ITEM	REPORTED ON PAGE #
		the included sources of evidence, as well as sources of funding for the scoping review. Describe the role of the funders of the scoping review.	**Funds for Health Improvement and Research (Grant No. 2022-1-2071)** and the **National Natural Science Foundation of China (Grant No. 82372386)**.

JBI = Joanna Briggs Institute; PRISMA-ScR = Preferred Reporting Items for Systematic reviews and Meta-Analyses extension for Scoping Reviews.
* Where *sources of evidence* (see second footnote) are compiled from, such as bibliographic databases, social media platforms, and Web sites.
† A more inclusive/heterogeneous term used to account for the different types of evidence or data sources (e.g., quantitative and/or qualitative research, expert opinion, and policy documents) that may be eligible in a scoping review as opposed to only studies. This is not to be confused with *information sources* (see first footnote).
‡ The frameworks by Arksey and O'Malley (6) and Levac and colleagues (7) and the JBI guidance (4, 5) refer to the process of data extraction in a scoping review as data charting.
§ The process of systematically examining research evidence to assess its validity, results, and relevance before using it to inform a decision. This term is used for items 12 and 19 instead of "risk of bias" (which is more applicable to systematic reviews of interventions) to include and acknowledge the various sources of evidence that may be used in a scoping review (e.g., quantitative and/or qualitative research, expert opinion, and policy document).

From: Tricco AC, Lillie E, Zarin W, O'Brien KK, Colquhoun H, Levac D, et al. PRISMA Extension for Scoping Reviews (PRISMAScR): Checklist and Explanation. Ann Intern Med. 2018;169:467–473. doi: 10.7326/M18-0850.
