# Supplementary material for: Clinical Applications and Emerging Roles of Bone Wax in Orthopaedic Surgery: A Scoping Review
Source: J Clin Med. 2026 Jul 3;15(13):5226. doi: 10.3390/jcm15135226 (PMC13363597; doi:10.3390/jcm15135226)
Supplement: Supplementary file 1 [file jcm-15-05226-s001.zip › jcm-4358662-supplementary/Supplementary File S2. Literature Searching Process.pdf]

Supplementary File S2. Search Strategies for Databases

This appendix presents the detailed search strategies used to identify relevant literature for this scoping review on the development and clinical applications of bone wax in orthopaedic surgery.

The searches were conducted across five major databases (PubMed, Embase, Ovid MEDLINE, Scopus, and Web of Science), supplemented by a gray literature search via Google Scholar.

All searches were limited to publications in English involving human studies from database inception to May 2025.

Boolean operators (AND, OR, NOT) and controlled vocabulary (MeSH/Emtree) were used to ensure both sensitivity and specificity.

1. Pubmed

#1 "bone wax"[MeSH Terms] OR "bone wax"[All Fields] OR "absorbable bone wax"[All Fields] OR "resorbable bone wax"[All Fields] OR "novel bone wax"[All Fields]
#2 "orthopaedic surgery"[MeSH Terms] OR "orthopedic procedures"[All Fields] OR "arthroplasty"[All Fields] OR "joint replacement"[All Fields] OR "arthroscopy"[All Fields] OR "spine surgery"[All Fields] OR "fracture fixation"[All Fields] OR "orthopaedic operation"[All Fields]
#3 #1 AND #2
#4 NOT ("animal experiment"[All Fields] OR "in vitro"[All Fields])
#3 Filters applied: English, Humans, Publication date from database inception to 2025/05/31
In total: 49

2. Embase

#1 ('bone wax'/exp OR 'bone wax' OR 'absorbable bone wax' OR 'resorbable bone wax' OR 'biodegradable bone wax' OR 'novel bone wax')
AND
#2 ('orthopedic surgery'/exp OR 'orthopaedic surgery' OR 'orthopedic procedure' OR 'arthroplasty'/exp OR 'arthroscopy'/exp OR 'spine surgery' OR 'fracture fixation'/exp)
#3 #1 AND #2
AND [english]/lim
AND [humans]/lim
AND [database inception-2025]/py
In total: 233

3. Ovid MEDLINE

#1. exp Bone Wax/ OR (bone wax OR absorbable bone wax OR resorbable bone wax OR novel bone wax).mp.
#2. exp Orthopedic Procedures/ OR (orthopaedic surgery OR orthopedic surgery OR arthroplasty OR arthroscopy OR spine surgery OR fracture fixation).mp.
#3. #1 AND #2
5. limit #3 to (english language and humans)
6. limit #3 to yr="database inception - 2025"
In total: 50

4. Scopus

TITLE-ABS-KEY (
("bone wax" OR "absorbable bone wax" OR "resorbable bone wax" OR "biodegradable bone wax" OR "novel bone wax")
AND
("orthopaedic surgery" OR "orthopedic procedures" OR "arthroplasty" OR "joint replacement" OR "arthroscopy" OR "spine surgery" OR "fracture fixation")
)
AND (LIMIT-TO (LANGUAGE, "English"))
AND (EXCLUDE (EXACTKEYWORD, "animal experiment") AND EXCLUDE (EXACTKEYWORD, "in vitro"))
In total: 112

5. Web of Science

TS = (
("bone wax" OR "absorbable bone wax" OR "resorbable bone wax" OR "biodegradable bone wax" OR "novel bone wax")
AND
("orthopaedic surgery" OR "orthopedic surgery" OR "arthroplasty" OR "joint replacement" OR "arthroscopy" OR "spine surgery" OR "fracture fixation")
)
Refined by:
[Language: (English)]
AND [Document Type: (Article OR Review)]
AND [Timespan: database inception-2025]
In total: 42

6. Google Scholar (Gray Literature)

"bone wax" AND ("hemostasis" OR "haemostasis" OR "hemostatic agent")
AND ("orthopaedic surgery" OR "arthroplasty" OR "arthroscopy" OR "spine surgery" OR "fracture fixation")
- patents - citations
Date range: database inception–2025
Language: English
