# Supplementary material for: Clinical Applications and Emerging Roles of Bone Wax in Orthopaedic Surgery: A Scoping Review
Source: J Clin Med. 2026 Jul 3;15(13):5226. doi: 10.3390/jcm15135226 (PMC13363597; doi:10.3390/jcm15135226)
Supplement: Supplementary file 1 [file jcm-15-05226-s001.zip › jcm-4358662-supplementary/Supplementary File S3. Inclusion and Exclusion Criteria.pdf]

Supplementary File S3. Inclusion and Exclusion Criteria

**Category**	**Criteria**	**Rationale**
Inclusion Criteria	1. Studies investigating the clinical use of bone wax in orthopaedic surgery or related procedures (e.g., arthroplasty, trauma fixation, spine surgery, arthroscopy).<br>2. Studies reporting composition, hemostatic effect, complications, or outcomes related to bone wax use.<br>3. Randomized controlled trials, cohort studies, retrospective observational studies, and case reports.<br>4. Articles published in English.<br>5. Publication period: from database inception to May 2025.	To ensure comprehensive coverage of all available clinical evidence relevant to the use and outcomes of bone wax in orthopaedic surgery.
Exclusion Criteria	1. Studies unrelated to bone wax or not describing its use in orthopaedic surgery.<br>2. Articles focused solely on non-orthopaedic applications (e.g., neurosurgery, thoracic surgery).<br>3. Preclinical, animal, or in-vitro material studies without clinical data.<br>4. Non-English publications, abstracts without full text, or duplicate reports.	To maintain a clear clinical focus and avoid confounding evidence from purely experimental or non-orthopaedic settings.
